# Efficacy and safety of 0.5% colchicine cream versus 5% 5-fluorouracil cream in the treatment of cutaneous field cancerization: a randomized clinical trial^⋆^

**DOI:** 10.1016/j.abd.2023.09.005

**Published:** 2024-04-12

**Authors:** Amanda Soares Teixeira, Ivanka Miranda de Castro Martins, Anna Carolina Miola, Hélio Amante Miot

**Affiliations:** a Department of Dermatology, Instituto Lauro de Souza Lima, Bauru, SP, Brazil; b Department of Infectology, Dermatology, Radiotherapy and Diagnostic Imaging, Faculty of Medicine, Universidade Estadual Paulista, Botucatu, SP, Brazil

**Keywords:** Colchicine, Fluorouracil, Keratosis, actinic, Randomized controlled trials as a topic, Skin neoplasms

## Abstract

**Background:**

5-Fluorouracil (5-FU) is a first-line drug to treat cutaneous field cancerization (CFC). There are few clinical trials with topical colchicine (COL).

**Objective:**

To evaluate the effectiveness of 0.5% COL cream *versus* 5% 5-FU cream in the treatment of CFC.

**Method:**

This was a randomized, open, self-controlled clinical trial. Forty-five patients (90 forearms), with three to ten actinic keratoses (AK) on each forearm, used 0.5% COL cream 2×/day for seven days on one forearm, and 5% 5-FU cream 2× /day, for 21 days, on the other forearm. The dosages were defined based on previous clinical trials for each drug. Adverse effects were evaluated after 14 days and outcomes after 90 days of inclusion. The primary outcome was complete AK clearance and the secondary outcomes were: partial clearance (≥50%), reduction in AK count, assessment of the Forearm Photoaging Scale (FPS), AK Severity Score (AKSS), and adverse effects.

**Results:**

After 90 days, there was complete clearance of AK in 37% (95% CI 24%–49%) and partial clearance in 85% (95% CI 76%–93%) of the forearms treated with 5-FU,*versus* 17% (95% CI 7%–27%) and 78% (95% CI 66%–88%) for COL (p > 0.07). There was a percentage reduction of 75% in the AK count of the forearms treated with 5-FU (95% CI 66%–83%) and 64% in those treated with COL (95% CI 55%–72%). Regarding FPS and AKSS, there was improvement in both groups, with no difference regarding FPS (p = 0.654), and 5-FU superiority for AKSS (p = 0.012).

**Study limitations:**

Single-center study.

**Conclusions:**

5-FU and COL are effective for treating CFC, with neither showing superiority regarding the reduction in AK counts.

## Introduction

Actinic keratoses (AK) are atypical proliferations of keratinocytes induced by radiation, especially ultraviolet radiation, resulting from chronic sun exposure.^1^ Classically, they are considered pre-malignant lesions, with a potential for progression to squamous cell carcinoma (SCC) of up to 0.075% per year for an individual lesion.^2^ Beyond the analysis of individual lesions, patients with multiple AK have a much higher cumulative incidence of SCC than patients without AK.^3^ Cutaneous field cancerization (CFC) is characterized by multiple AK and/or SCC *in situ*, occurring in an area chronically exposed to ultraviolet radiation.^4^

AK and CFC treatment aims to improve patient quality of life, eradicate lesions, prevent the emergence of cutaneous malignancies and their recurrence.^5^ Therapeutic options can be directed to treatment of individual lesions (destructive therapies) or CFC.^6^, ^7^ As the atypia that predisposes the emergence of malignant neoplasms occurs even in areas without clinically visible lesions, it is recommended to treat the entire CFC and, in this context, topical treatments are the most frequently used options.

5-Fluorouracil (5-FU) is a pyrimidine analogue with antitumor action first described in 1957,^8^ and has been used for over 50 years to treat pre-malignant and malignant skin lesions.^9^ Its action occurs mainly through the inhibition of thymidylate synthase and, as it interferes with the cell replication process, cells with a high mitotic index are more susceptible to its cytotoxic effect, while normal cells seem to be much less sensitive to these effects.^10^ Local adverse reactions are an impediment to complete adherence to treatment, which usually lasts from 14 to 28 days.^11^

Colchicine (COL) is one of the oldest drugs still currently used. It is a natural alkaloid extracted from the corm of the plant *Colchicum autumnale* and other species of the genus *Colchicum*.^12^ Its use in the treatment of AK was described in 1968 by Marshall and is based on the antimitotic action of the drug, as it impairs the composition of microtubules and cell division. In 2000, the first clinical trial that evaluated the effectiveness of COL for treating AK showed a total clearance of 70% in AK on the scalp of patients treated with COL after 60 days.^13^

The search for new treatment options, which may offer greater tolerance, adherence, efficacy, and/or safety than existing ones, is desirable. Although decades have passed since the first reports of its use for the treatment of AK, there are few published clinical trials that allow establishing the efficacy and safety of COL for the treatment of AK and CFC. Moreover, there are no studies comparing COL to 5-FU in the treatment of CFC. This study, therefore, aims to evaluate the efficacy of 0.5% COL cream *versus* 5% 5-FU cream in the treatment of CFC in immunocompetent patients with multiple AK on the forearms.

## Methods

This was a randomized, open, self-controlled clinical trial, comparing 0.5% COL cream *versus* 5% 5-FU cream, carried out at the Dermatology Outpatient Clinic of Instituto Lauro de Souza Lima (ILSL) in Bauru, state of São Paulo, Brazil. The study was approved by the institutional ethics committee (n. 3,751,592), registered in the Brazilian Registry of Clinical Trials (https://ensaiosclinicos.gov.br/rg/RBR-487ctp), and financed by the Dermatology Support Fund (FUNADERM, *Fundo de Apoio à Dermatologia*).

After agreeing to participate and signing the consent form, 45 immunocompetent patients were included, of both genders, over 18 years old and with a clinical diagnosis of three to ten AK on each forearm, who had not undergone any treatments for AK or CFC in the last six months.

The established exclusion criteria were: extensive dermatoses affecting one of the forearms, known hypersensitivity or allergy to any of the assessed substances, immunosuppression due to medications and/or diseases, pregnancy, breastfeeding and use of retinoids, anti-inflammatories or any other topical treatment in the evaluated region.

The participants were randomized to define which forearm would receive 5-FU or COL, in a 1:1 allocation, generated by computer simulation (block randomization). Randomization was performed by a researcher not involved in patient evaluation, using sequential allocation, whereas patient inclusion and evaluation were carried out by another researcher. Each individual underwent two treatments, one on each forearm. 5-FU was applied twice daily for 21 days and COL twice daily for seven days. The patients also received sunscreen for regular use on both forearms throughout the study period. The application of medications and sunscreen was carried out by the patients themselves.

At inclusion, each participant received a package corresponding to their randomization group, containing: (1) Three 15 g tubes of 5% 5-FU cream (Efurix®, Valeant); (2) One airless light-resistant bottle of 20 g (release of 1 g per pump) of 0.5% COL cream (compounded product, Pharmácia Specífica Ltda.); (3) Two 200 mL tubes of sunscreen (Neutrogena® Sun Fresh FPS30 Body Sunscreen); (4) One sheet with instructions for use, contact telephone number and illustration showing the area and side on which each medication should be used, in addition to the method of use according to the randomization group. All medication packages were labeled as “right” or “left” (with an illustration of the corresponding forearm) and the method of use for each drug, following forearm randomization, to reduce the risk of confounding treatments.

**Fig. 1:**
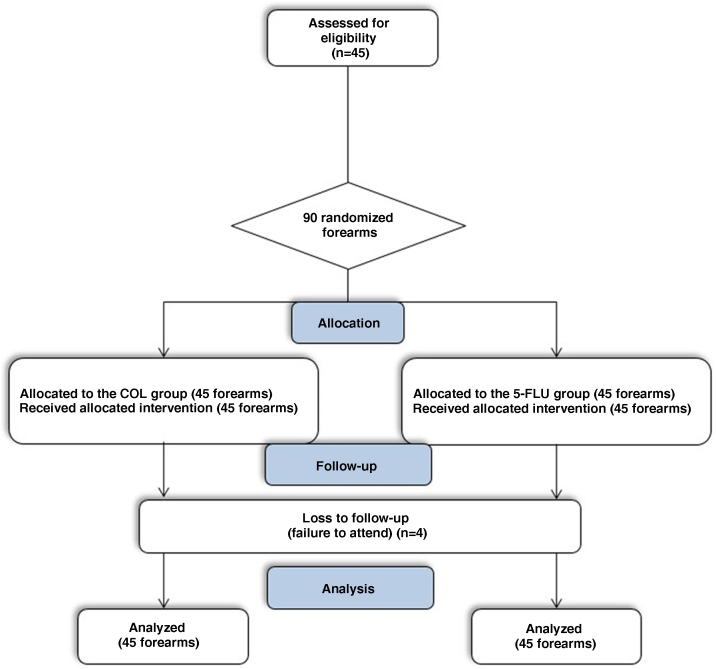
CONSORT flowchart of the study.

For each patient, the study lasted 90 days, with three assessments: (1) D0 ‒ Inclusion, AK count on each forearm, Forearm Photoaging Scale (FPS) and AK severity score(AKSS); (2) D14 ‒ assessment of tolerability and adverse effects; (3) D90 ‒ AK count, FPS and AKSS (Fig. 1).

**Fig. 2:**
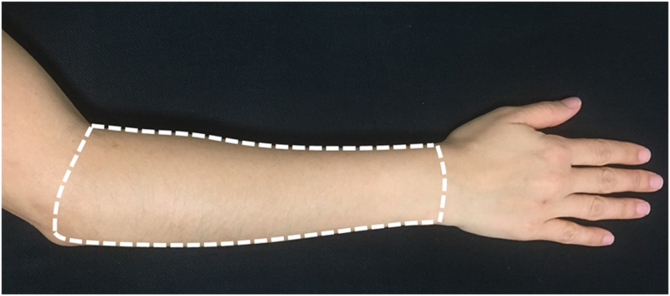
Representation of the area submitted to treatments ‒ dorsal side of the forearm (Source: Researchers archive).

For evaluation and treatment, the dorsal surface of each forearm was defined as the area delimited by an imaginary line connecting the styloid processes of the ulna and radius, the medial border of the forearm, the lateral border of the forearm, and a line joining the epicondyle lateral to the antecubital fossa (Fig. 2).

After marking the KA with a pen, the KA count was performed and confirmed for each forearm, by the same evaluator (A.S.T.) in both assessments.

The FPS is a validated scale that measures the degree of aging of the forearms through the number of superficial and hypertrophic AK, quantity and severity of wrinkles, solar lentigines, visible purpura, stellar atrophic scars, elastosis, and loss of elasticity. Each criterion has a weight and, at the end of the evaluation, the values are added together, resulting in a number that varies from 0 to 192.^14^

The AKSS allows for assessing the response to therapeutic modalities for CFC in a qualitative way, being complementary to the AK count. Diameter in millimeters, degree of hyperkeratosis, and exulceration of each AK are the considered parameters, and the final AKSS value is given by the sum of the scores of all AK in the assessed area.^15^

The primary assessed outcome was the rate of complete clearance of AK in each forearm. Secondary outcomes were partial clearance (≥50% reduction in AK count), reduction in AK count, improvement in FPS, reduction in AKSS, and assessment of adverse effects.

Comparing adverse effects caused by topical therapies with different dosages, in the same patient, can be complex, since the peak of local reactions occurs at different times. COL cream had its use evaluated, in most studies, on a twice-a-day schedule for ten days.^16^, ^17^, ^18^ Previous clinical trials with topical COL carried out by the authors research group showed that the last days of treatment led to more marked local reactions.^16^, ^17^ In the present study, a shorter seven-day course was adopted, aiming to assess whether the reduction of the treatment period would demonstrate the same efficacy, but with fewer adverse effects. Otherwise, the usual dose of 5% 5-FU cream for the treatment of AK is twice a day for two to four weeks, with adverse effects being more intense the longer the therapeutic regimen.^19^ Considering the above, a seven-day treatment course was established for COL, a 21-day treatment for 5-FU, and the assessment of adverse effects was carried out 14 days after the start of treatments, a moment considered adequate to not overestimate the occurrence of local reactions due to each therapy.

The sample was sized to detect a 30% or higher difference in the complete clearance rate of AK (D90) between groups (e.g., 16% vs. 46%). A power of 0.85 was adopted; an alpha of 0.05 and an estimated dropout rate of up to 20%, resulting in 45 patients (90 forearms).^20^

All patients included in the study and randomized were part of the ITT (intention to treat) population. AK clearance, FPS, and AKSS of the forearms were compared by time and groups (over time) using the generalized linear mixed effects model, robust covariance structure, type 1 autoregressive matrix covariance, Šidák posthoc comparison, and gamma probability distribution or negative binomial adjustment when indicated. The missing cases had their values imputed by the mixed model.

The effect size was calculated as the difference in the means of each variable on D0 and D90, and the confidence interval using the bootstrap technique with 1000 resamples. The data were analyzed using IBM SPSS 25v. Significance was set as p < 0.05.^21^

## Results

From May 2021 to April 2022, 45 eligible patients were included and underwent treatment with 5% 5-FU cream on one forearm and 0.5% COL cream on the contralateral forearm (total of 90 forearms).

The main clinical and demographic data of the 45 participants at inclusion (D0) are shown in Table 1. There was a predominance of women (62%), the mean age of the participants was 67.3 years, phototype II was the most common (69%) and most patients had a personal history of skin cancer (60%) and previous intense sun exposure (64%).

**Table 1:** Demographic data of all 45 participants at the inclusion in the study (D0).

Variables			Values	
Gender, n (%)				
	Male		17	(38%)
	Female		28	(62%)
Age, in years				
	Mean		67.3	
	SD		10.4	
Phototype, n (%)				
	II		31	(69%)
	III		14	(31%)
Level of schooling, n (%)				
	Illiterate		4	(9%)
	Elementary School		29	(64%)
	High School		10	(22%)
	Higher Education		2	(4%)
Previous sun exposure, n (%)				
	No exposure		0	(0%)
	Mild		0	(0%)
	Moderate		16	(36%)
	Intense		29	(64%)
Current sun exposure n (%)				
	No exposure		10	(22%)
	Mild		19	(42%)
	Moderate		7	(16%)
	Intense		9	(20%)
Leisure with sun exposure, n (%)			12	(27%)
History of skin cancer, n (%)			27	(60%)
Current smoking, n (%)			9	(20%)
Counting of AK^a^			5 (4−6)^b^	
FPS^a^			55 (42−75)^b^	
AKSS^a^			7 (5−10)^b^	

AK, Actinic Keratosis; FPS, Forearm Photoaging Scale; AKSS, actinic keratosis severity score.

a Per forearm.

b Median (p25‒p75).

Regarding the clinical outcomes, 15 forearms treated with 5-FU showed complete AK clearance on D90 (37%; 95% CI 24%–49%), whereas among those treated with COL, seven achieved this outcome (17%; 95%CI 7%–27%; p = 0.077). Partial clearance (≥50% reduction in the number of AK) was achieved by 35 of the forearms treated with 5-FU (85%; 95% CI 76%–93%) and 32 of those treated with COL (78%; 95% CI 66%–88%; p = 0.508).

On D90, there was a percentage reduction of 75% in AK count for the forearms treated with 5-FU (95% CI 66%–83%) and 64% for those treated with COL (95% CI 55%–72%), with improvement over time for both treatments (p < 0.05), albeit with no difference between them (p = 0.069). None of the participants showed worsening AK counts.

**Fig. 3:**
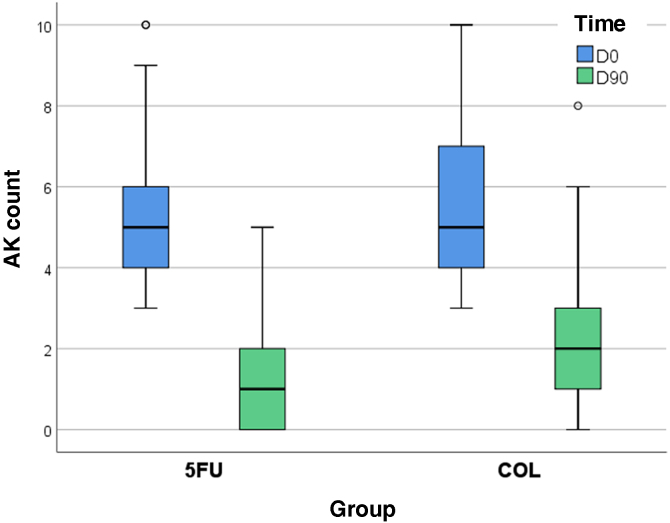
Distribution of actinic keratosis counts on the forearms on D0 and D90, comparing treatments.

The intergroup difference regarding FPS improvement was not relevant either (p = 0.654); on the other hand, both treatments led to a significant reduction in AKSS, and in this regard, 5-FU was superior (p = 0.012; Table 2, Fig. 3).

**Table 2:** Main clinical outcomes comparing the two forearms.

Variables				5-FU	COL	p-value
Complete clearance			15 (37%; 95%CI 24%–49%)		7 (17%; 95%CI 7%–27%)	0.077
Partial clearance			35 (85%; 95%CI 76%–93%)		32 (78%; 95%CI 66%–88%)	0.508
AK counting						
	D0		5 (4–6)		5 (4–7)	
	D90		1 (0–2)^b^		2 (1–3)^b^	0.069
	Percentage reduction		75% (66%–83%)		64% (55%–72%)	
Photoaging scale						
	D0		55 (43–75)		55 (42–76)^a^	
	D90		44 (36–64)^b^		45 (36–67)^b^	0.654
AK severity scale						
	D0		7(5-10)		7(5-10)	
	D90		1 (0–2)^b^		2 (1-4)^b^	**0.012**#

a Median (p25-p75)

b (p<0,05) D0 *vs*. D90

# p < 0,05

Regarding adverse effects, this study showed that COL caused more pain than 5-FU, in addition to causing more edema and triggering more intense desquamation. In contrast, 5-FU led to greater crust formation (Table 3). However, only one participant (2.2%) reported not being willing to undergo the treatments again if necessary.

**Table 3:** Main adverse effects of treatments on D14 (n = 43).

Variables		5-FU	COL	p-value
Pain		1 (1–3)^a^	1 (1–5)^a^	**0.018**#
Irritation				0.169
	None	5 (12%)	6 (14%)	
	Mild	22 (51%)	15 (35%)	
	Moderate	11 (26%)	10 (23%)	
	Severe	5 (12%)	12 (28%)	
Erythema				0.217
	Absent	8 (19%)	9 (21%)	
	Mild	26 (61%)	20 (47%)	
	Moderateo	8 (19%)	9 (21%)	
	Intense	1 (2%)	5 (12%)	
Edema				**0.035**#
	Absent	41 (95%)	34 (79%)	
	Mild	2 (5%)	8 (19%)	
	Moderate	- (-)	1 (2%)	
Desquamation				**0.005**#
	Absent	22 (51%)	14 (33%)	
	Mild	17 (40%)	17 (40%)	
	Moderate	3 (7%)	8 (19%)	
	Intense	1 (2%)	4 (9%)	
Bullae				0.125
	Absent	43 (100%)	39 (91)	
	Mild	- (-)	3 (7%)	
	Moderate	- (-)	1 (2%)	
Crusts				**<0.001**#
	Absent	5 (12%)	11 (26%)	
	Mild	23 (54%)	30 (70%)	
	Moderate	13 (30%)	1 (2%)	
	Intense	2 (5%)	1 (2%)	

a Median (p25‒p75).

# p < 0,05

No malignant neoplasms appeared in the treated regions during the 90-day follow-up. During the study period, there were four dropouts, so 41 participants (82 forearms) underwent all three assessments (D0, D14, and D90). The dropouts did not occur due to adverse effects of the intervention, but rather due to patients not showing up on the day scheduled for the evaluation, and the impossibility of rescheduling (Fig. 1). Missing participants were contacted and did not attend for reasons linked to the COVID-19 pandemic.

## Discussion

The present study showed no difference regarding AK treatment efficacy between 5-FU 5% and COL 0.5% in the tested doses and duration of treatment. Since 5-FU is an established and strongly recommended therapy for treatment of AK and CFC,^7^ this information also reinforces COL as a therapeutic option, with an affordable cost and a dose that facilitates adherence, despite more intense adverse effects.

Among the forearms treated with 5-FU, 37% showed complete AK clearance, a result very similar to that found in a double-blind randomized clinical trial, with 932 patients, where the effectiveness of 5% 5-FU cream for six months was evaluated. following a twice-daily course of treatment for four weeks on the face and ears (38% complete clearance with 5-FU *versus* placebo 17%).^22^

Another randomized clinical trial, involving 602 patients, which compared 5-FU 5% to three other treatment options for AK on the head, showed clearance ≥75% in 90.6% of patients three months after the end of treatment.^23^ Similarly, the present study found 85% partial clearance (≥50%) in the forearms treated with 5-FU.

Regarding the treatment of AK in the upper limbs, a self-controlled clinical trial compared the use of 5% 5-FU cream twice a day in two 15-day cycles separated by a 15-day break *versus* four fortnightly sessions of 70% glycolic acid peeling + 5% 5-FU solution, indicating an 85.7% reduction in the number of AK in the limbs treated with 5-FU cream.^24^ The smaller sample size of the aforementioned study, the treatment area including the hands, as well as the different doses, which may interfere with adherence to treatment, are characteristics that may explain the slightly favorable difference in relation to the 75% reduction in AK count found in the present study.

Complete clearance of 17% was observed in the forearms submitted to a seven-day course of 0.5% COL cream twice daily. The same rate was found in a study that used 0.5% COL in the forearms twice a day for ten days in 36 assessed forearms.^16^ These data suggest that a shorter course of seven days may be equally effective in improving AK and CFC. It is not possible to infer results resulting from monthly, sequential courses.

A more recent clinical trial comparing COL (twice a day for seven days) *versus* ingenol mebutate or sunscreen, associated or not with oral *Polypodium leucotomos*, showed complete clearance, after 60 days, of 14.7% for COL, 16.1% for ingenol mebutate and 2.8% for sunscreen.^17^ The present study and the two aforementioned clinical trials show a similar clearance rate for COL, which demonstrates its consistency as a treatment option for CFC.^16^, ^17^

The authors found a 64% reduction in AK counts in the forearms treated with COL when comparing D0 with D90. A previous study showed a superior result for COL in the upper limbs, with a reduction of 73.4%.^18^ This superiority can be justified by the fact that, in the aforementioned study, a second course of treatment lasting ten days was carried out in patients who showed little or no inflammatory response to the first course.

The first clinical trial that tested the use of COL for AK treatment compared the application of COL gel twice a day for ten days to placebo, showing complete resolution of the lesions in seven of the ten treated patients, while none of the patients showed a response in the placebo group. In contrast to the present study, this clinical trial was not controlled, there was no washout period before inclusion (an unknown number of participants had undergone other treatments for AK shortly before the study), the concentration of COL used was 1% in gel vehicle, the treated area was the forehead and the sample was considerably smaller,^13^ differences that may justify the discrepancy in the complete clearance rate.

There are no industrialized topical presentations of COL, but it is possible to obtain topical formulations containing COL through manipulation pharmacies. Regarding solubility, 1 g of COL dissolves in 22 mL of water, 220 mL of ether, and 100 mL of benzene, freely in alcohol or chloroform and is practically insoluble in petroleum ether or petroleum jelly.^25^ Non-ionic cream was used as a vehicle in the present study, as it has a good solubility profile for COL, allowing drug penetration. Two previous clinical trials^16^, ^17^ carried out by this research group used the same formulation, produced by the same manipulation pharmacy (Pharmácia Specífica Ltda), achieving very similar clearance rates, which suggests constancy and stability of the formulations. COL darkens when exposed to light,^26^ which is why light-resistant, airless bottles were chosen for storing COL cream.

Regarding treatment of forearm photoaging, 5-FU has been previously shown to improve the visual appearance of the skin, in addition to increasing the expression of pro-collagen I in the evaluation carried out six months after treatment.^27^ Both treatments investigated in the present study led to improvement in FPS. This result makes one reflect on whether COL could also bring benefits in photoaging treatment, raising the importance of studies with a design dedicated to elucidating this issue.

AKSS was the outcome that showed a significant superiority of 5-FU in relation to COL (p = 0.012). The criteria that define this scale are the diameter of the lesions, degree of hyperkeratosis, and presence of ulceration.^15^ It is important to consider that, for the patient, hyperkeratosis and ulceration improvement, even if there is no complete lesion resolution, can bring more satisfaction and comfort.

Relevant differences were found in relation to the adverse effects of the treatments: COL caused more pain, more edema, and more desquamation than 5-FU; on the other hand, 5-FU caused greater crust formation. The occurrence of adverse reactions may be a reason for the patient not to adequately adhere to treatment; however, the main factor that compromises adherence to topical treatments for AK and CFC is the prolonged treatment duration.^28^ Therefore, even though COL is more often associated with some adverse effects, the fact that it has a shorter course of treatment compared to 5-FU makes it an advantageous treatment option for AK and CFC.

A study that evaluated skin reactions resulting from the use of 5% 5-FU twice a day for four weeks showed that intense erythema was the most frequent reaction (46.7%), followed by pruritus (28.9%). %), burning sensation (21.5%), and desquamation (18.5%).^11^ The present study showed erythema in 82% and desquamation in 49% of the forearms treated with 5-FU. However, in the present study, erythema was classified as intense in only 2% of cases. The difference can be explained by the fact that the aforementioned study determined the occurrence of adverse effects through telephone reports from the participants, while in the present study, reactions were evaluated through dermatological examination.

Furthermore, the additional role of sunscreens in the treatment of CFC should not be neglected. A study with a similar population that included 40 individuals (80 forearms) with AK on the forearms, showed a reduction of approximately 30% in the number of lesions after 60 days.^29^

The limitations of the present study include the relative homogeneity of the studied population, as it is a single-center study.

It is extremely valid that the investigation of other COL dosages schedules are performed aimed at identifying more effective and comfortable forms of use. It is also pertinent that more comparative studies be carried out with COL in other areas, such as the face and scalp.

## Conclusions

5-FU and COL are effective for treating CFC, with complete and partial clearance rates that showed no statistical difference after 90 days. 5-FU was superior in reducing the severity of AK. COL caused more pain, edema, and desquamation than 5-FU, but the latter resulted in more crust formation.

## Financial support

Dermatology Support Fund of Brazilian Dermatology Society (FUNADERM, *Fundo de Apoio à Dermatologia*).

## Authors' contributions

Amanda Soares Teixeira: Project design; collection of data; review of the literature; writing and approval of the manuscript.

Ivanka Miranda de Castro Martins: Collection of data; writing and approval of the manuscript.

Anna Carolina Miola: Project design; review of the literature; writing and approval of the manuscript.

Hélio Amante Miot: Project design; analysis of data; review of the literature; writing and approval of the manuscript.

## Conflicts of interest

None declared.
